# The Akt/mTOR pathway in cancer stem/progenitor cells is a potential therapeutic target for glioblastoma and neuroblastoma

**DOI:** 10.18632/oncotarget.26088

**Published:** 2018-09-11

**Authors:** Hisham F. Bahmad, Tarek H. Mouhieddine, Reda M. Chalhoub, Sahar Assi, Tarek Araji, Farah Chamaa, Muhieddine M. Itani, Amaly Nokkari, Firas Kobeissy, Georges Daoud, Wassim Abou-Kheir

**Affiliations:** ^1^ Department of Anatomy, Cell Biology and Physiological Sciences, Faculty of Medicine, American University of Beirut, Beirut, Lebanon; ^2^ Department of Biochemistry and Molecular Genetics, Faculty of Medicine, American University of Beirut, Beirut, Lebanon; ^3^ Current Address: Department of Medical Oncology, Dana-Farber Cancer Institute, Harvard Medical School, Boston, MA, USA; ^4^ Current Address: Medical Scientist Training Program, College of Medicine, Medical University of South Carolina, Charleston, SC, USA

**Keywords:** glioblastoma, neuroblastoma, rapamycin, triciribine, cancer stem cell

## Abstract

Nervous system tumors represent some of the highly aggressive cancers in both children and adults, particularly neuroblastoma and glioblastoma. Many studies focused on the pathogenic role of the Akt pathway and the mechanistic target of Rapamycin (mTOR) complex in mediating the progression of various types of cancer, which designates the Akt/mTOR signaling pathway as a master regulator for cancer. Current studies are also elucidating the mechanisms of cancer stem cells (CSCs) in replenishing tumors and explicating the strong correlation between the Akt/mTOR pathway and CSC biology. This instigates the development of novel treatments that target CSCs via inhibiting this pathway to prevent recurrence in various cancer subtypes. In accordance, neuroblastoma and glioblastoma tumors are believed to originate from stem/progenitor cells or dedifferentiated mature neural/glial cells transformed into CSCs, which warrants targeting this subpopulation of CSCs in these tumors. In our study, Triciribine and Rapamycin were used to assess the role of inhibiting two different points of the Akt/mTOR pathway *in vitro* on U251 (glioblastoma) and SH-SY5Y (neuroblastoma) human cell lines and their CSCs. We showed that both drugs minimally decrease the survival of U251 and SH-SY5Y cells in a 2D model, while this effect was much more pronounced in a 3D culture model. Triciribine and Rapamycin decreased migratory abilities of both cell lines and decreased their sphere-forming units (SFU) by extinguishing their CSC populations. Together, we concluded that Rapamycin and Triciribine proved to be effective in the *in vitro* treatment of glioblastoma and neuroblastoma, by targeting their CSC population.

## INTRODUCTION

Cancers of the central nervous system (CNS) are devastating medical conditions that claimed the lives of an estimated number of 15,320 patients in 2015 worldwide [1]. One of the most pertinent CNS tumors is glioblastoma multiforme, which has one of the poorest prognoses and highest chance of recurrence due to its diffusely infiltrative capabilities, with an annual incidence rate of 2–3 per 100,000 in Europe and North America [2]. Another prevalent form of tumors of nervous origin is neuroblastoma, a neural crest tissue-derived tumor, which represents one of the most commonly diagnosed tumors under 1 year of age, with an incidence rate of 10.2 per million children being affected below 15 years of age [3].

In addition to radiotherapy and surgical interventions, current medical treatments of CNS tumors include a wide array of chemotherapeutic agents including alkylating agents, alkaloids, mechanistic target of Rapamycin (mTOR) inhibitors, antibiotics and monoclonal antibodies. Current cancer research is investigating alternative targets of treatment in order to improve prognosis and achieve long-term remission. Recently, novel therapies are being developed to specifically target the cancer stem cells (CSC), a sub-population of self-regenerating cells with tumorigenic potential, found in different cancer subtypes [4, 5] and believed to be the reason behind tumor recurrence for exhibiting resistance to current therapeutic modalities.

Rapamycin, also known as Sirolimus, is a macrolide and an inhibitor of the PI3K/Akt/mTOR pathway, a pathway that is often deregulated in cancer [6], which in turn has given Rapamycin potent anti-cancerous properties in a variety of solid tumors and blood-related malignancies [7]. On the other hand, Triciribine is a tricyclic purine nucleoside, which is metabolically activated into its active monophosphate analogue that prevents the phosphorylation and subsequent activation of Akt [8]. The targeted pathway has been shown to be aberrantly activated in both Glioblastoma [9] and Neuroblastoma [10] primary human samples, remarkably showing an increase in the levels of phosphorylated Akt in the cytoplasm of the malignant cells.

This pathway is driven upstream by a receptor tyrosine kinase (RTK), responsible of activating phosphoinositide 3-kinase (PI3K), an upstream kinase responsible of the phosphorylation of several downstream effector molecules, including Akt, at Thr308. mTOR complexes are activated downstream of the AKT pathway [10]. Rapamycin blocks the ability of mTOR complex 1 to auto-phosphorylate, inhibiting its ability to activate downstream molecules, like p70S6 kinase (p70S6K), responsible of driving forward the cell cycle. [9] The Akt/mTOR pathway is summarized in Supplementary Figure 1.

Both drugs have been studied in various types of cancers, with Triciribine being studied to a lesser extent compared to Rapamycin [11–14]. Yet, since each of the mTOR and Akt pathways have been implicated in both glioblastoma [9, 15] and neuroblastoma [10, 16], we decided to investigate the efficacy of both drugs in the progression of cancers of nervous origin by studying their effects on two cancer cell lines, namely the glioblastoma U251 and neuroblastoma SH-SY5Y human cell lines. Our main interest was to study the effect of inhibition of the mTOR and Akt pathways in the CSC population of these cell lines via a sphere-forming assay within Matrigel™ [17–20]. Similar to our previously described experiment [17], the inhibitory effects of Rapamycin and Triciribine on sphere-formation were studied over five consecutive generations of spheres, to investigate the efficacy of these drugs in potentially suppressing the CSC population within the tumor bulk.

## RESULTS

### U251 and SH-SY5Y cell proliferation is inhibited by rapamycin and triciribine

**Figure 1:**
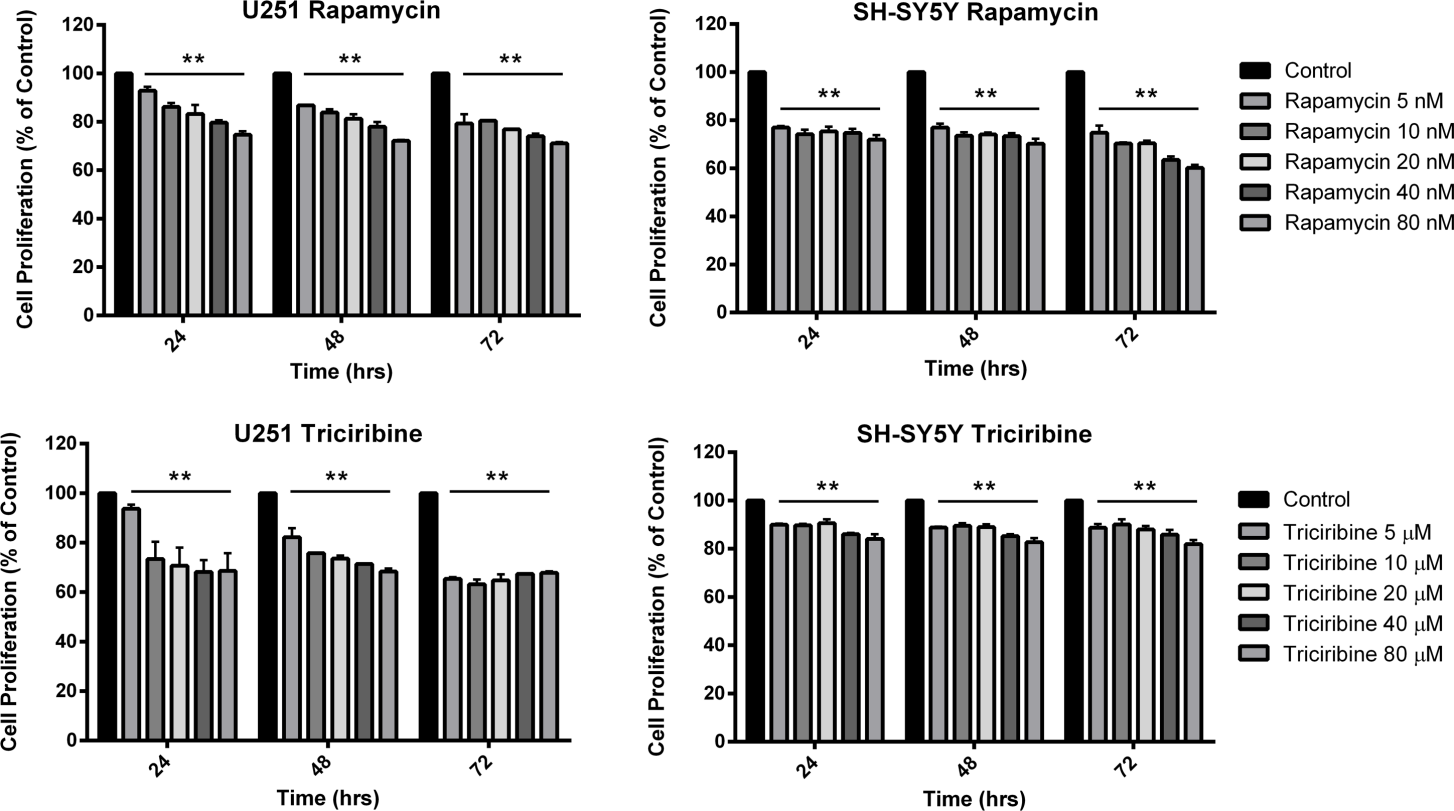
The effect of various concentrations of Rapamycin and Triciribine on the proliferation of U251 and SH-SY5Y cell lines After incubation of the two cell lines (U251 and SH-SY5Y) for 24, 48 and 72 hr with or without treatment with Rapamycin or Triciribine of increasing concentrations, cell proliferation was determined using MTT assay. Results are expressed as a percentage of the treated group compared to its control. Data represent an average of three independent experiments. The data are reported as mean ± SEM (*p < 0.001*; One-way ANOVA; ^**^P < 0.01; different treatment concentrations compared to control, Tukey’s multiple comparison test).

The *in vitro* effect of Rapamycin and Triciribine on the cell proliferation of U251 and SH-SY5Y was assessed using the MTT assay (Figure 1). Both drugs had significant anti-proliferative effects on both cell lines. Nonetheless, the metabolic activity of the treated cells didn’t decrease to less than 60% of that of the untreated cells (control) at any time point, irrespective of the treatment concentration of both drugs. As noticed, the inhibitory effects of both drugs reached a plateau-like state with insignificant alterations between different combination of concentration at several time points. While the effect of Rapamycin was almost the same on both cell lines, the inhibitory effect of Triciribine was more pronounced on U251 compared to SH-SY5Y.

### Rapamycin and triciribine inhibit the migratory ability of cancer cell lines

**Figure 2:**
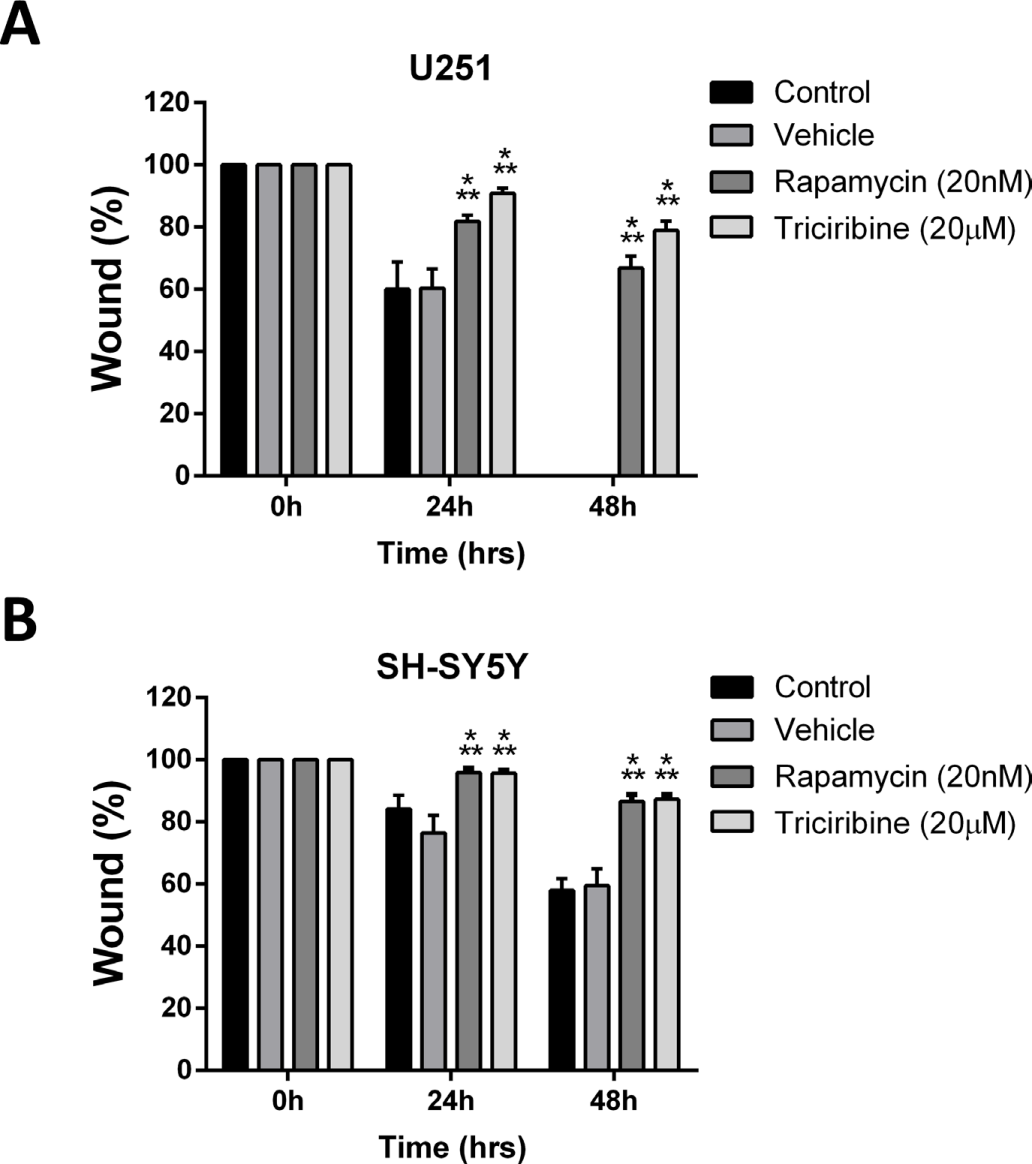
Rapamycin and Triciribine reduce the migratory potentials of human neuronal and glial cancer cell lines A scratch was made in a six-well plate of confluent U251 and SH-SY5S cells using a 200 μl tip, and images were taken at *T* = 0, 24, and 48 h with or without treatment, and quantification of the distance of the wound closure was assessed over time (**A**, **B**). Results are expressed as a percentage of each group compared to its condition at *T* = 0 h. Data represent an average of three independent experiments. The data are reported as mean ± SEM (*p < 0.001*; Two-way ANOVA; ^***^P < 0.001; different treatments compared to control at this time point, Bonferroni’s multiple comparison test).

The effect of both drugs on migration index of both cell lines was studied using the scratch/wound healing assay. Mitomycin C, a cellular proliferation inhibitor, was used prior to the wound formation on U251 cell line exclusively. Addition of Mitomycin C on SH-SY5Y was omitted as it led to their death. Rapamycin and Triciribine significantly suppressed the wound closure of U251 cells by 65% and 78%, respectively, compared to the untreated cells which were able to migrate and completely close the wound at 48 hours (Figure 2A). Rapamycin and Triciribine had similar effects on SH-SY5Y cell lines, significantly inhibiting 87% of wound closure at 48h, whereas untreated cells were successfully able to close up to 50% of the wound at the same time point. (Figure 2B). These data support the potential ability of Rapamycin and Triciribine to contain the metastatic ability of both cancer cell lines.

### Rapamycin and triciribine inhibit akt and mtor pathways respectively

**Figure 3:**
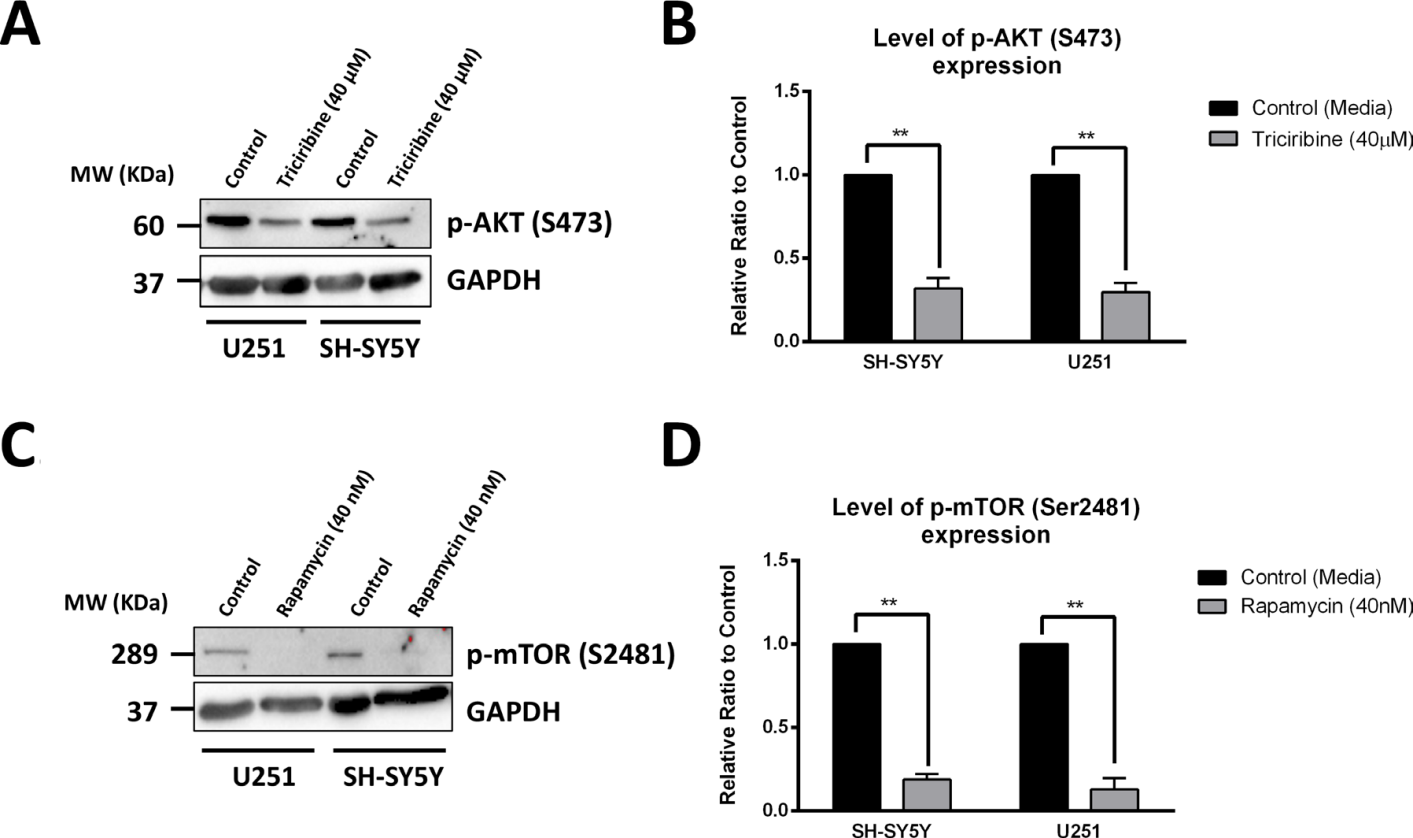
Rapamycin and Triciribine selectively inhibit the autophosphorylation of mTOR and Akt, respectively After treating SH-SY5Y and U251 cells with 40 μM Triciribine and 40 nM Rapamycin for 48 hours, proteins were extracted using RIPA buffer, and used to detect differences in expression of the phosphorylated form of AKT (S473) and mTOR (S2481), respectively. Bands were detected by enhanced chemiluminescence (ECL) using ChemiDoc MP Imaging System (**A**, **C**). Protein expression was quantified using Image Lab software, relative to the expression of GAPDH, a housekeeping gene equally expressed in treated and non-treated cells. Results are expressed as relative ratio to control (**B**, **D**). Data represent an average of three independent experiments. The data are reported as mean ± SEM (^**^P < 0.01; treatment compared to control, *t*-test).

The direct effects of Triciribine and Rapamycin, on their respective targets Akt and mTOR, were assessed using western blot, to detect differences in protein expression between the cellular lysates of treated and non-treated U251 and SH-SY5Y cells. Akt inhibition by Triciribine was established by monitoring the levels of expression of the activated form of Akt, phosphorylated at Serine 473 (p-Akt S473). Treating cells with Triciribine significantly decreased the expression of p-Akt (S473) in both SH-SY5Y and U251 cell lines by 68% (*p* = 0.0084) and 70% (*p* = 0.0058), as compared to the control group, respectively (Figure 3A and 3B).

The effect of Rapamycin on specifically targeting mTOR was assessed by probing for the autophosphorylation product of the active mTOR at Serine 2481. As expected, treating cells with Rapamycin significantly decreased the expression of the p-mTOR (S2481) in SH-SY5Y and U251 cell lines by 81% (*p* = 0.0015) and 87% (*p* = 0.0059), respectively (Figure 3C and 3D).

The total level of expression of Akt and mTOR was further investigated as baseline control for the effects of both drugs. No significant change in the level of expression of both proteins was noticed after treatment with Triciribine and Rapamycin. (Supplementary Figure 2).

### Rapamycin and Triciribine target an enriched population of U251 and SH-SY5Y cancer stem/progenitor cells

**Figure 4:**
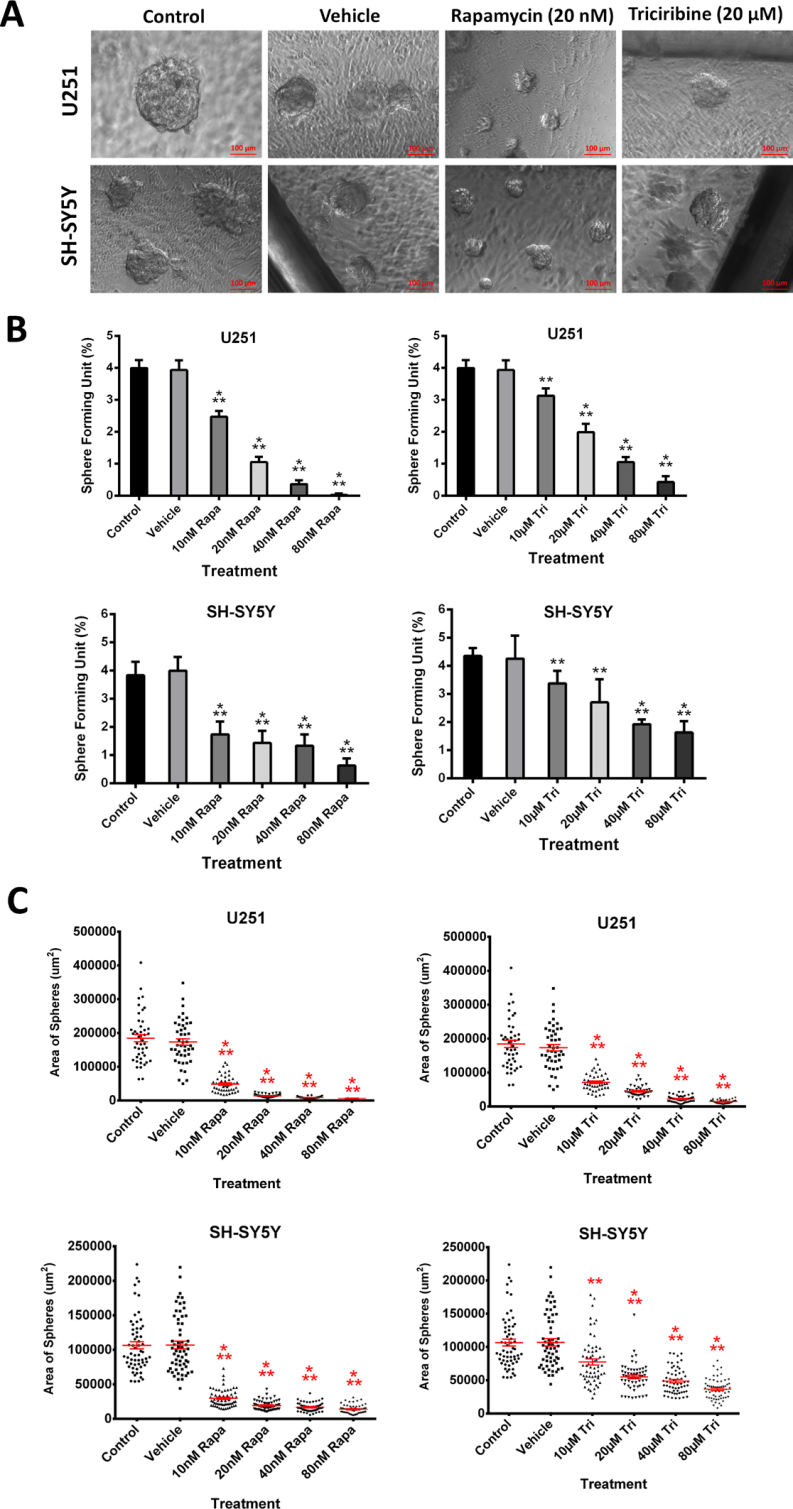
Rapamycin and Triciribine abolish sphere-forming ability of human neuronal and glial cancer cell lines (**A**) Representative bright-field images of U251 and SH-SY5Y spheres with or without Rapamycin and Triciribine treatment. Images were visualized by Axiovert inverted microscope at 10× magnification and analyzed by Carl Zeiss Zen 2012 image software. (**B**) Quantification of the average size of U251 and SH-SY5Y spheres with or without treatment conditions. Data represent an average area (μm^2^) of 20–30 measured spheres. The data are reported as mean ± SEM (*p < 0.001*; One-way ANOVA; ^***^P < 0.001; different treatment concentrations compared to control, Tukey’s multiple comparison test). **(C)** Increasing the concentration of treatment of both Rapamycin and Triciribine on U251 and SH-SY5Y spheres, cultured in Matrigel™, led to a similar decrease in the number of sphere-forming units (SFU) in both cell lines. The generated spheres are referred to as G1 (Generation 1) spheres. Results are expressed as SFU which is calculated according to the following formula: SFU = (number of spheres counted × number of input cells) × 100. Data represent an average of three independent experiments. The data are reported as mean ± SEM (*p < 0.001*; One-way ANOVA; ^***^P < 0.001; different treatment concentrations compared to control, Tukey’s multiple comparison test).

The sphere-forming capability was studied by culturing single cell suspensions of both U251 and SH-SY5Y in Matrigel™ for 9 and 14 days, respectively. The obtained spheres were visualized under an inverted light microscope (Figure 4A). Compared to SH-SY5Y, the U251 cell line produced larger spheres (Figure 4A and 4C).

We finally studied the inhibitory effect of increasing dose concentrations of both drugs on the cancer stem/progenitor cell populations in U251 and SH-SY5Y cell lines via sphere-forming assay. We generated the spheres from 2,000 single cells embedded in Matrigel™ and obtained a sphere-forming unit (SFU) of around 4% for both U251 and SH-SY5Y cells (Figure 4B). Both drugs led to a significant decrease in the size (Figure 4C) and in the number of spheres formed in a dose- dependent manner (Figure 4B), though more prominent with Rapamycin. It was clear that the U251 cell line was more susceptible to the drugs compared to the SH-SY5Y cell line, whereby the SFU of U251 was decreased by around 75% at a concentration of 20 nM of Rapamycin and 40 μM of Triciribine. On the other hand, 80 nM of Rapamycin and 80 μM of Triciribine were needed to decrease the SFU of SH-SY5Y neurospheres to around 85% and 63%, respectively. Interestingly, when comparing the effect of the two drugs on the same cell line, Rapamycin had a relatively higher inhibitory effect.

Notably, when compared to 2D culture, Rapamycin and Triciribine were more potent when used in a 3D culture, whereby the inhibitory effect on 2D-cultured cells reaching a plateau as observed in the MTT experiment wasn’t present when we checked the inhibitory effects of the same drugs on 3D-cultured neuro- and gliospheres, showing a dose-dependent decrease in the proliferative ability of both cell lines at lower drug dosages.

**Figure 5:**
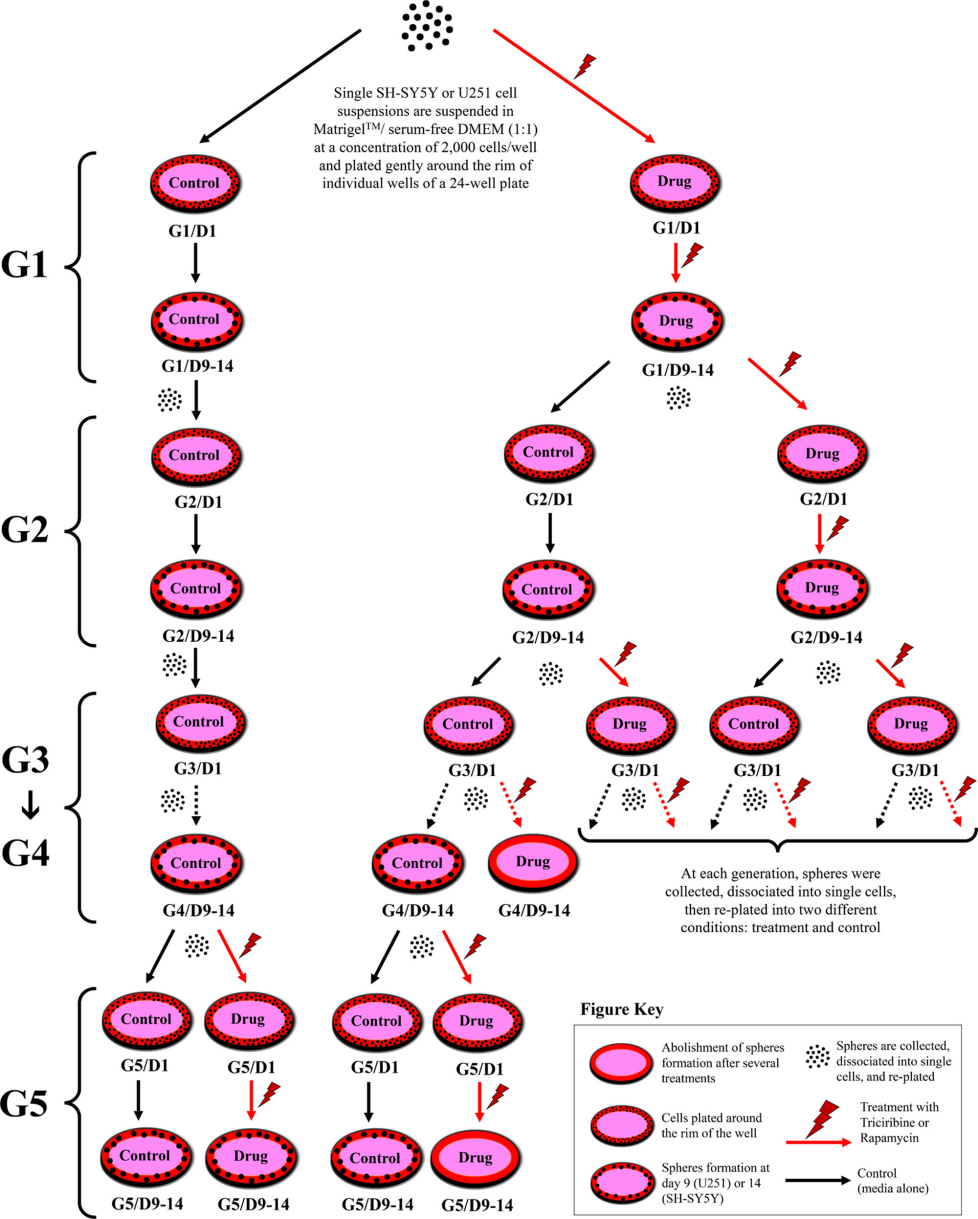
Schematic diagram demonstrating strategy of drug treatments in sphere-formation assay 2 × 10^3^ of U251 or SH-SY5Y cell suspensions are suspended in Matrigel™/serum free DMEM (1:1) and plated gently around the rim of individual wells of a 24-well plate. Media (alone or with drug) is added gently to the center of each well and changed every 2–3 days. Spheres are formed and serially passaged without treatment for 5 generations (G) to enrich for cancer stem cells, and then drugs are added at G5 to potentially target those cells; or treatment is added to every generation and then spheres are serially propagated to investigate whether the effect is permanent or reversal. The SFU, expressed as %, is calculated by dividing the number of spheres counted by the number of input cells (2 × 10^3^ cells) and then multiplied by 100.

**Figure 6:**
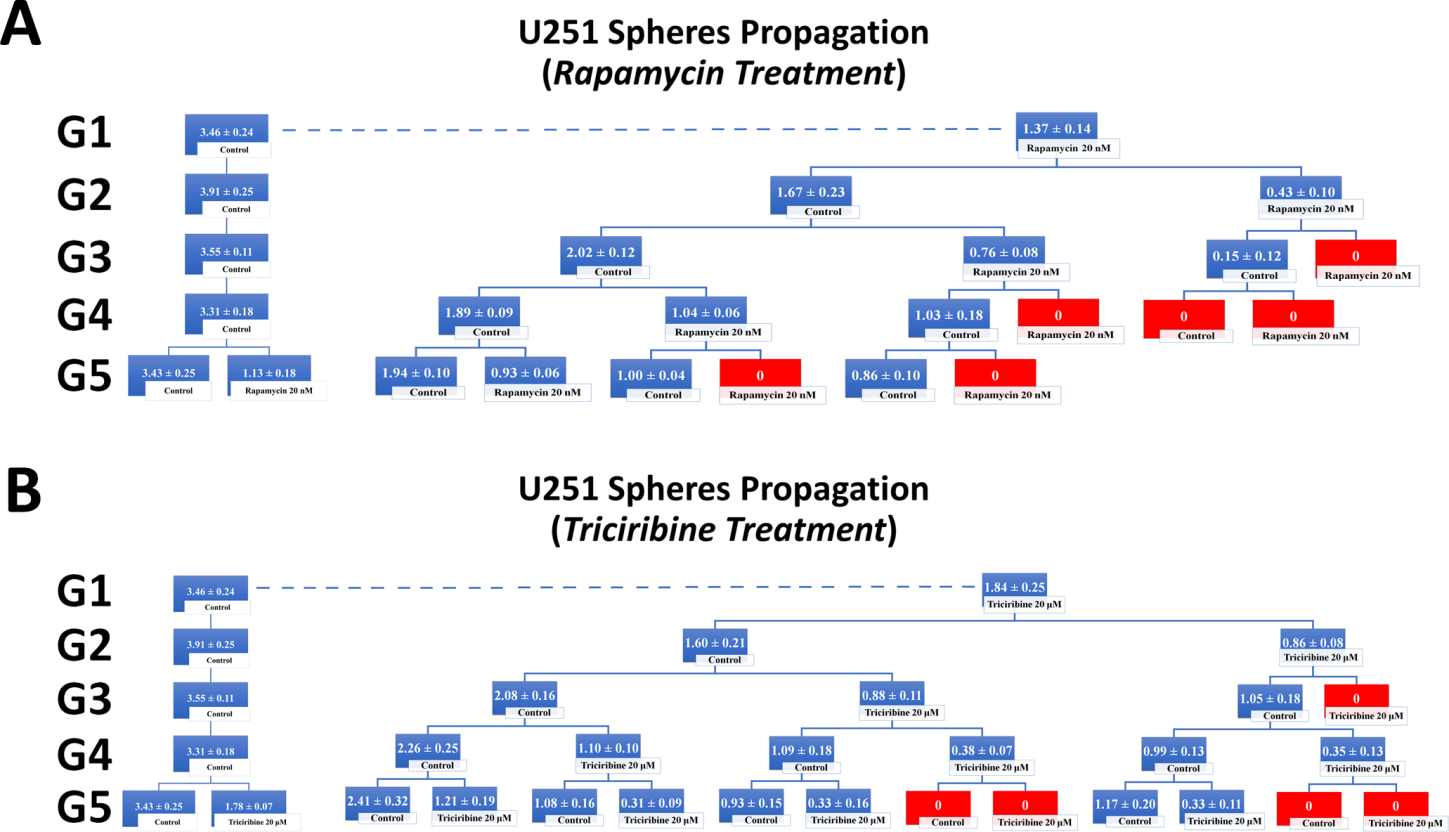
Rapamycin and Triciribine inhibit the self-renewal ability of the glioblastoma U251 cancer stem/progenitor cells SFU obtained from serially passaged U251 gliospheres over five generations is shown under untreated conditions (taken as control) and with 20 nM Rapamycin and 20 μM Triciribine-treated condition (treated once at G5) (left panels of charts **A** and **B**). Glioblastoma CSC were enriched from U251 cell line and treated with either Rapamycin (20 nM) (A) or Triciribine (20 μM) (B). After each propagation, cells that were initially treated with Rapamycin, Triciribine or media (control) were seeded into separate wells and exposed to control or treatment conditions (right panels of charts A and B). Spheres were propagated for five generations and results are expressed as SFU. Data represent an average of three independent experiments. The data are reported as mean ± SEM.

**Figure 7:**
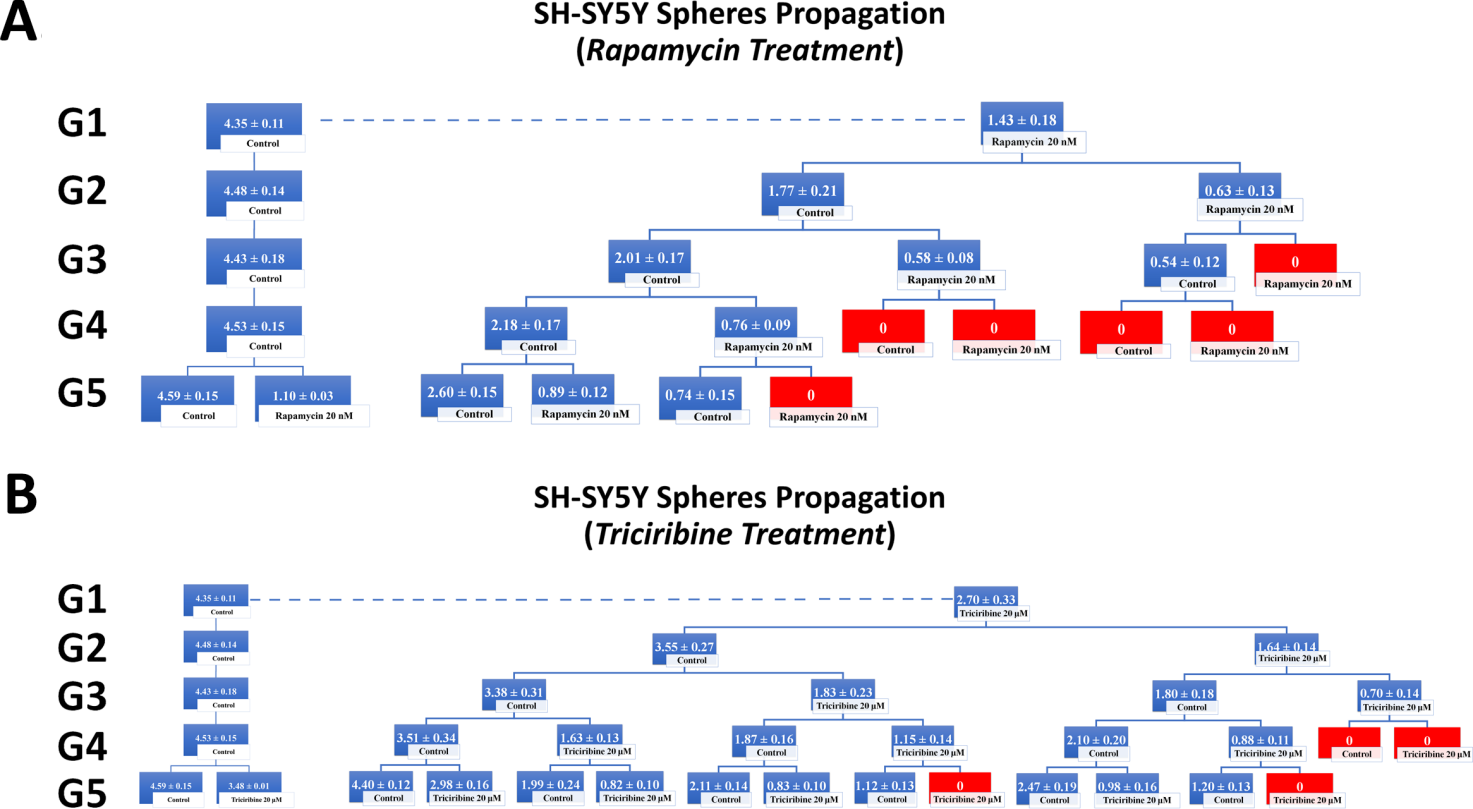
Rapamycin and Triciribine inhibit the self-renewal ability of the neuroblastoma SH-SY5Y cancer stem/progenitor cells SFU obtained from serially passaged SH-SY5Y neurospheres over five generations is shown under untreated conditions (taken as control) and with 20 nM Rapamycin and 20 μM Triciribine-treated condition (treated once at G5) (left panels of charts **A** and **B**). Neuroblastoma CSC were enriched from SH-SY5Y cell line and treated with either Rapamycin (20 nM) (A) or Triciribine (20 μM) (B). After each propagation, cells that were initially treated with Rapamycin, Triciribine or media (control) were seeded into separate wells and exposed to control or treatment conditions (right panels of charts A and B). Spheres were propagated for five generations and results are expressed as SFU. Data represent an average of three independent experiments. The data are reported as mean ± SEM.

To investigate the self-renewal ability of stem/progenitor cells, we assessed the effect of Rapamycin and Triciribine on the sphere forming capability of U251 and SH-SY5Y cells over five generations. After collecting the spheres of the first generation (G1), they were dissociated into single cells and propagated by reseeding the same number of cells used in G1 (2 × 10^3^ cell/well). Figure 5 represents a schematic diagram demonstrating the experimental design of this assay and Figures 6 and 7 illustrate the drug treatments on U251 and SH-SY5Y spheres formation, respectively, upon propagation over 5 consecutive generations.

Treating the generated U251 gliospheres of the first generation (G1) with Rapamycin (20 nM) and Triciribine (20 μM) resulted in significant decrease in the sphere-forming units of 60% and 45% respectively (Figure 6). The same drug concentrations led to an approximate decrease of 65% (with 20 nM Rapamycin) and 40% (with 20 μM Triciribine) in the sphere-forming units of SH-SY5Y cells at G1 (Figure 7). These results were consistent with those we got after propagating untreated U251 and SH-SY5Y cells over 5 generations and treating them once at G5 with Rapamycin and Triciribine (Left panels of charts in Figures 6 and 7). The overall potency of Rapamycin was much compelling as compared to Triciribine when propagating U251 and SH-SY5Y cells over five generations, reaching an SFU of 0% upon two consecutive or alternative treatments with Rapamycin (Right panels of charts in Figures 6 and 7). Noteworthy, following treatment withdrawal of both drugs, we noticed a stable SFU percentage in the first treatment-free generation, followed by an increase in the subsequent generations. This argues for the consecutive or alternative treatments needed to eradicate any population of cancer stem/progenitor cells.

## DISCUSSION

The treatment of tumors of nervous origin remains a great challenge in the clinical setting due its low response degree, complications and high mortality rate. Many signaling pathways have been implicated in the initiation and progression of cancer and have thus become potential therapeutic targets in oncolysis. Among those pathways, the PI3K/Akt/mTOR/S6K1 pathway has gained a huge interest, given its pivotal role in driving oncogenesis [7, 21, 22]. Previous studies discussed the important role of this pathway in the development and oncogenesis of glioblastoma [23] and neuroblastoma [10].

Triciribine’s target of inhibition Akt, also known as protein kinase B (PKB), is a serine/threonine kinase that has three isolated isoforms: Akt1 (PKBα), Akt2 (PKBβ) and Akt3 (PKBγ), whereby Akt1 is specifically up-regulated in most cancers [24]. Upon Akt phosphorylation, there is a downstream activation of targets promoting cell survival, including mTOR, and inhibition of pro-apoptotic proteins, including Bcl-2 associated death promotor (Bad) and p53 [25]. Furthermore, mTOR activation, namely via mTOR complex 1 (mTORC1), leads to the subsequent activation of a group of proteins required for cell cycle progression such as cyclin D [26] and inhibition of pro-apoptotic proteins such as eukaryotic translation elongation factor 2 kinase (eEF-2K) [27] and cyclin-dependent kinase inhibitors such as p27kip1 [28]. On the other hand, mTORC2 is thought to be necessary for cellular metabolism, cytoskeletal regulation, Akt activation and is not inhibited by Rapamycin [29]. However, it was shown that prolonged treatment with Rapamycin in specific cell types might indeed prevent the assembly of mTORC2 and in turn down-regulate Akt signaling [30]. Besides, treating with low concentrations of Rapamycin (0.5–100 nM) revealed specific mTORC1 targeting, while higher concentrations (0.2–20 μM) have been shown to target mTORC2 as well [31]. On the other hand, Triciribine exhibits growth inhibition at concentrations as low as 1–10 µM by inhibiting phosphorylation of Akt, as well as its downstream p70S6K at basal levels reaching 100 µM [32].

Gursel *et al.* studied the importance of the AKT/mTOR signaling pathway, downstream of the epidermal growth factor receptor (EGFR) using mouse-derived astrocytic primary cell lines, showing high similarity to glioblastoma from human origin [32]. This study showed different responses of the generated glial cell lines to the inhibitory effects of several drugs, including Triciribine and Rapamycin. To our interest, Rapamycin inhibition revealed incomplete ability to inhibit growth of astrocytic malignant cell lines, as compared to Akt inhibitor, Triciribine [32]. Therefore, the mTOR-AKT pathway provides different targets for specific chemotherapeutic agents, with a differential response across different tumors depending on the molecular target itself, as well as the cancer type. Moreover, studies investigated the role of Akt and mTOR in maintaining the integrity of CSCs in different malignancies, including glioblastoma and neuroblastoma. It was found that inhibiting mTOR [33–35] or Akt [36] decreased the pool of CD133^+^ cells, which exhibit CSC properties in specific cancers, including gliomas. Treating cells with Rapamycin, a specific inhibitor of mTOR, showed a decrease in the proliferation and viability of the entire glial cell populations, including stem/progenitor cells: the percentage of CD133+ cells remained after treatment in pancreatic cancers [35]. That study points out that targeting the mTOR pathway may be a successful method to destroy CD133^+^ CSCs, which exhibit resistance to conventional radio- and chemotherapy. Nevertheless, other studies showed that mTOR inhibition in gastrointestinal cancer cells and liver tumor cells could actually lead to an increase in the number of CD113^+^ cells [37, 38]. On the other hand, Bleau *et al.* demonstrated that Akt, but not its downstream target mTOR, regulates ATP binding cassette transporters (ABCG2) activity, which provides chemoresistance in glioma tumor stem-like cells [39].

Our study revealed that both Rapamycin and Triciribine significantly inhibit the proliferation and survival of two cell lines of glioblastoma and neuroblastoma. Even though Triciribine works at an upstream target of the pathway, thereby hypothesizing that it could be more potent, but less specific, than Rapamycin by inhibiting a wider range of cellular processes, it turns out that both drugs have comparable effects. That could be explained by the superior importance of mTOR pathway, as compared to other molecular targets covered by the upstream AKT pathway: while rapamycin directly inhibits the activation of mTOR, Triciribine inhibits upstream processes responsible of mTOR activation, and thus converging in their downstream effects. Furthermore, this effect could be also explained by the effect of Rapamycin in inhibiting mTORC2, after prolonged treatment, subsequently leading to the down-regulation of Akt.

Additionally, we showed that the Akt/mTOR pathway is essential for U251 and SH-SY5Y migration. The Akt/mTOR signaling cascade could thus be a target of adjunct anti-cancer therapy whereby using Akt or mTOR inhibitors could hinder disease progression and dissemination while undergoing radio and/or chemotherapy, thereby greatly improving prognosis.

Finally, we studied the effect of inhibiting Akt and mTOR on the CSC population within the U251 and SH-SY5Y cell lines, using Matrigel™-based 3D spheres culturing assay. Several studies have validated that response to treatment as well as the gene expression profiles upon culturing cells in 3D spheroid models resemble more the physiological *in vivo* environment of the human body [40–42]. In a recent study by Riedl *et al.*, authors showed that although 3D models of colon cancer-derived spheroids showed significant decrease in the level of activity of the AKT-mTOR pathway as compared to 2D models, they significantly augmented the anti-tumor response as compared to their 2D counterparts [40]. Their findings emphasized on the importance of 3D spheroid models as a more valid system in the development and testing of anti-cancer agents *in vitro*. Moreover, cancer therapy is currently in the process of developing anti-malignant agents able to specifically target CSC, with the increasing evidence showing their function in tumorigenesis and tumor recurrence. Our results revealed that inhibiting either Akt or mTOR via Triciribine and Rapamycin, respectively, would lead to a similar decrease in sphere formation ability in both glioblastoma and neuroblastoma cell lines. Furthermore, both drugs were also able to eradicate the self-renewal ability of U251 CSCs with subsequent treatment with both drugs to reach an SFU of 0% by the third generation. However, after two generations of treatment withdrawal we noticed that U251 cells regained some of their sphere forming ability and thus had an increased SFU again, which we were able to inhibit again with another treatment cycle. Therefore, it is pertinent to serially treat the cancerous cells with either Rapamycin or Triciribine to eliminate the CSC population. Interestingly, both drugs exhibited a higher potency when being used in 3D culture models compared to the 2D cultures, where cell growth inhibition reached a plateau with increasing drug concentrations. Therefore, Triciribine and Rapamycin, as inhibitors of the AKT/mTOR pathway, may be further studied for their potential use as adjunct therapy in the treatment of neuroblastoma and glioblastoma, given their *in vitro* ability to successfully inhibit and eradicate an enriched population of CSC.

In conclusion, our study further supports the notion that the Akt/mTOR pathway is a fundamental element in the progression of nervous system tumors and that targeting it via Rapamycin and/or Triciribine showed promising results in suppressing the proliferation, migration, invasion and self-replication of cell line models of these tumors. Knowing that Rapamycin has made it into clinical trials of different cancers, Triciribine appears to be also worthy of consideration. More importantly, these two drugs should be targeting the CSC population of cancers via successive treatments in order to eradicate the subsequent generations of CSCs. Future studies should address the safety of using these drugs in terms of being cytotoxic toward primary healthy cells and their use in combination with other cytotoxic drugs that kill the non-CSCs.

## STUDY LIMITATIONS

This work builds on previous and current recent studies from our and other labs that evaluate the effect of different Akt and mTOR inhibitors on neuroblastoma and glioblastoma tumors [17, 43–45]. Yet, some limitations are related to the study methodology and experimental design. For example, our work was based on assessing the inhibitory effect of both drugs on one human cancer cell line as a model for each of the neuroblastoma and glioblastoma tumors. Consequently, more future experiments will follow after acquiring more human cell line models for both tumors to test the inhibitory effect of both drugs using different cell lines. Also, as majority of studies on cancer stem cells are done *in vivo*, we are planning to expand our experiments to *in vivo* studies as well.

## MATERIALS AND METHODS

### Cell culture and treatments

U251 and SH-SY5Y cells (ATCC, USA) were cultured and maintained in Dulbecco’s Modified Eagle Media (DMEM) Ham’s F-12 (Sigma-Aldrich) supplemented with 10% heat inactivated fetal bovine serum (FBS) (Sigma-Aldrich) and 1% Penicillin/Streptomycin (Sigma-Aldrich). Cells were incubated at 37° C in a humidified incubator containing 5% CO_2_. The drugs Triciribine and Rapamycin were purchased from Sigma-Aldrich and were both reconstituted in dimethyl sulfoxide (DMSO), per manufacturer’s instructions.

### MTT/Cell viability assay

The anti-proliferative effects of Triciribine and Rapamycin were measured *in vitro* by using MTT ([3-(4, 5-dimethylthiazol-2-yl)-2, 5-diphenyltetrazolium bromide]) assay according to the manufacturer’s instructions (Roche). Briefly, cells were seeded (1 × 10^4^ cells/well) in 100 µl complete medium in three different 96-well plates - one plate per time point (24 hr, 48 hr, 72 hr) - and incubated overnight at 37° C, 5% CO_2_ before being exposed to the different treatments. At each time point, media was removed and replaced by fresh media along with 10 µl/well of the MTT yellow dye. The cells were incubated for 4 hr, after which 100 µl of the solubilizing agent was added to each well. The plate was incubated overnight at 37° C, 5% CO_2_. Absorbance intensity was measured by the microplate ELISA reader (Multiscan EX) at 595 nm. The percentage of cell viability was presented as an optical density (OD) ratio of the treated to the untreated cells.

### Wound healing assay

SH-SY5Y and U251 cells were cultured in 6-well plates (5 × 10^5^ cells/well) and incubated at 37° C, 5% CO_2_ until they reached 90–100% confluence. Cells were then treated with 10 mg/ml of Mitomycin C (Sigma) for 2 hr in order to block cellular proliferation. A sterile 200 µl tip was used to create scratch wounds of the same width on each monolayer. The plates were then washed twice with phosphate-buffered saline (PBS) to remove the detached cells, and the remaining cells were cultured in complete media with or without treatment. Photos were taken at 0, 24, and 48 hr, and the distance traveled by the cells enumerated the closure of the wounds.

### Western blotting

SH-SY5Y and U251 cells were cultured in 6-well plates (5 × 10^5^ cells/well) and incubated at 37° C, 5% CO_2_ until they reached 70% confluence. Three wells were randomly selected and treated with Rapamycin (40 nM) or Triciribine (40 μM) for 48 hours, while the remaining wells were taken as control. The plates were then washed with PBS to remove any residual media. Adherent cells were treated with RIPA buffer (0.1% SDS, 0.5% Sodium deoxylate, 150 mM NaCl, 1 mM EDTA, 50 mM TRIS-HCl (pH = 8), 1% NP40, protease and phosphatase inhibitors), scraped off the plates, transferred into micro-centrifuge tubes and incubated on ice for 30 minutes. Sonication was used to maximize the protein yield. Lysates were then centrifuged at 13,600 rpm for 20 minutes at 4° C, to pellet the cell debris.

Protein quantification was performed using the Detergent Compatible (DC) Protein Assay (from Bio-Rad) as per manufacturer’s recommendations, with serial dilutions of bovine serum albumin (BSA) taken as standards. Aliquots of proteins of equal amounts (50 μg) were mixed with sample buffer (with 5% β-mercaptoethanol) and separated on 8% SDS-PAGE gel. Proteins were transferred into Polyvinylidene difluoride (PVDF) membrane, which were blocked using 5% BSA in Tris-buffered Saline (TBS) with 0.1% Tween-20. The blots were incubated overnight at 4° C with specific rabbit primary antibodies in TBS with 5% BSA, targeting: phosphorylated mTOR (Ser2481) (Cell Signaling, 1:1000) or phosphorylated AKT (Ser473) (Cell Signaling, 1:1000). The membranes were later incubated with horseradish peroxidase (HRP) conjugated anti-rabbit secondary antibody (Santa Cruz, 1:2500), and bands were detected by enhanced chemiluminescence (ECL) using ChemiDoc MP Imaging System (BioRad). Glyceraldehyde 3-phosphate dehydrogenase (GAPDH) (Novus Biologicals, 1:2500) was used as a loading control.

### 3D culture and sphere-formation assay

Single SH-SY5Y and U251 cell suspensions were suspended in Matrigel™/serum free DMEM (1:1) at a concentration of 2 × 10^3^cells/well in a total volume of 50 µl. The solution was then plated gently around the rim of individual wells of a 24-well plate and allowed to solidify for 1hr at 37° C in a humidified incubator containing 5% CO_2_. 0.5 ml of DMEM with 2% FBS (for U251) or 5% FBS (for SH-SY5Y) was added gently to the center of each well and the media (containing the treatment) was changed every 2–3 days. Spheres were counted and/or harvested at day 9 (for U251) or day 14 (for SH-SY5Y) after plating. For sphere propagation, the medium was aspirated and the Matrigel™ was digested with 0.5 ml Dispase solution (Invitrogen, Carlsbad, CA, 1 mg/ml, dissolved in serum-free DMEM Ham’s F-12) for 60 minutes at 37° C. Spheres were collected, incubated in 1 ml warm Trypsin- EDTA at 37° C for 5 minutes, and then passed through a 27-gauge syringe 5 times. Cells were counted by a hemocytometer and re-seeded at 2 × 10^3^cells/well. Figure 7 represents a schematic demonstrating the experimental design of this assay. The sphere-forming unit (SFU), expressed as %, was calculated by dividing the number of spheres counted by the number of input cells (2 ×10^3^ cells) and then multiplied by 100. Zeiss Axiovert microscope was used for the acquisition of bright field images.

### Data analyses

Statistical analysis was performed using GraphPad Prism 6 analysis software. The significance of the data was analyzed using the student’s *t*-test, one-way ANOVA followed by Tukey’s Multiple comparison test, or Two-way ANOVA followed by Bonferroni’s Multiple comparison test. *P*-values of *p* < 0.01 (^**^) and *p* < 0.001 (^***^) were considered significant and highly significant, respectively.

## SUPPLEMENTARY MATERIALS FIGURES



## Abbreviations

mTOR: mechanistic target of Rapamycin
CSCs: cancer stem cells
SFU: sphere-forming unit
CNS: central nervous system
PKB: protein kinase B
mTORC1: mTOR complex 1
eEF-2K: eukaryotic translation elongation factor 2 kinase
FBS: fetal bovine serum
MTT: [3-(4,5-dimethylthiazol-2-yl)-2, 5-diphenyltetrazolium bromide]
OD: optical density
PBS: phosphate-buffered saline
PFA: paraformaldehyde
DC: Detergent Compatible
