# Supplementary material for: The Akt/mTOR pathway in cancer stem/progenitor cells is a potential therapeutic target for glioblastoma and neuroblastoma
Source: Oncotarget. 2018 Sep 11;9(71):33549–61. doi: 10.18632/oncotarget.26088 (PMC6173359; doi:10.18632/oncotarget.26088)
Supplement: Supplementary file 1 [file oncotarget-09-33549-s001.pdf]

The Akt/mTOR pathway in cancer stem/progenitor cells is a potential therapeutic target for glioblastoma and neuroblastoma

SUPPLEMENTARY MATERIALS

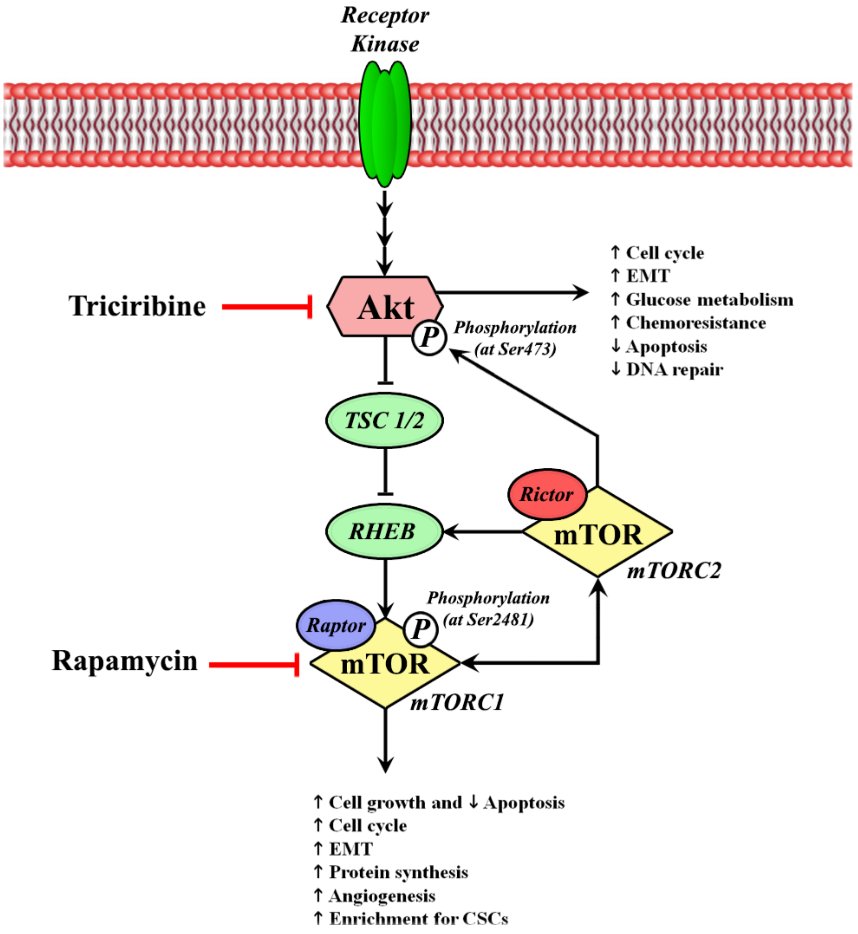

Supplementary Figure 1: Schematic representation of the Akt/mTOR signaling pathway and levels of inhibition by Triciribine and Rapamycin.

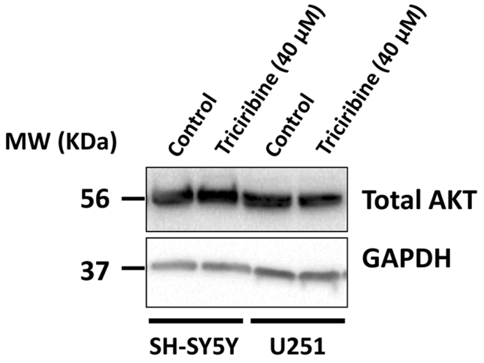

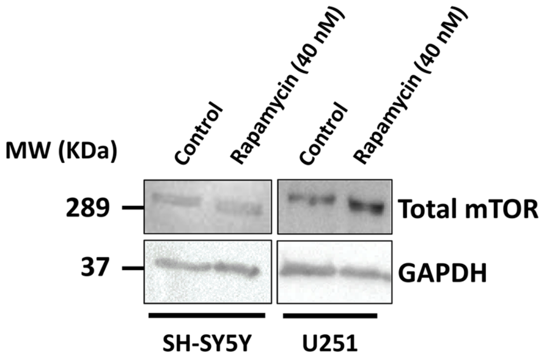

Supplementary Figure 2: Rapamycin and Triciribine display no effect on total mTOR and Akt protein expression levels, respectively. After treating SH-SY5Y and U251 cells with 40 µM Triciribine and 40 nM Rapamycin for 48 hours, proteins were extracted and used to detect differences in expression levels of total AKT and mTOR proteins, respectively. Bands were detected by enhanced chemiluminescence (ECL) using ChemiDoc MP Imaging System. Results represent three independent experiments.
